# Targeted RNAseq assay incorporating unique molecular identifiers for improved quantification of gene expression signatures and transcribed mutation fraction in fixed tumor samples

**DOI:** 10.1186/s12885-021-07814-8

**Published:** 2021-02-04

**Authors:** Chunxiao Fu, Michal Marczyk, Michael Samuels, Alexander J. Trevarton, Jiaxin Qu, Rosanna Lau, Lili Du, Todd Pappas, Bruno V. Sinn, Rebekah E. Gould, Lajos Pusztai, Christos Hatzis, W. Fraser Symmans

**Affiliations:** 1 Departments of Pathology and Translational Molecular Pathology, The University of Texas MD Anderson Cancer Center, Houston, TX USA; 2 Yale Cancer Center, Yale School of Medicine, New Haven, CT USA; 3 Department of Data Science and Engineering, Silesian University of Technology, Gliwice, Poland; 4 The Jackson Laboratory, Farmington, CT USA; 5 Delphi Diagnostics, Austin, TX USA; 6 Institute of Pathology, Charité Universitätsmedizin Berlin, Berlin, Germany

**Keywords:** Targeted RNAseq, Unique molecular identifiers, Assay development, Breast cancer

## Abstract

**Background:**

Our objective was to assess whether modifications to a customized targeted RNA sequencing (RNAseq) assay to include unique molecular identifiers (UMIs) that collapse read counts to their source mRNA counts would improve quantification of transcripts from formalin-fixed paraffin-embedded (FFPE) tumor tissue samples. The assay (SET4) includes signatures that measure hormone receptor and PI3-kinase related transcriptional activity (SET_ER/PR_ and PI3Kges), and measures expression of selected activating point mutations and key breast cancer genes.

**Methods:**

Modifications included steps to introduce eight nucleotides-long UMIs during reverse transcription (RT) in bulk solution, followed by polymerase chain reaction (PCR) of labeled cDNA in droplets, with optimization of the polymerase enzyme and reaction conditions. We used Lin’s concordance correlation coefficient (CCC) to measure concordance, including precision (Rho) and accuracy (Bias), and nonparametric tests (Wilcoxon, Levene’s) to compare the modified (NEW) SET4 assay to the original (OLD) SET4 assay and to whole transcriptome RNAseq using RNA from matched fresh frozen (FF) and FFPE samples from 12 primary breast cancers.

**Results:**

The modified (NEW) SET4 assay measured single transcripts (*p*< 0.001) and SET_ER/PR_ (*p*=0.002) more reproducibly in technical replicates from FFPE samples. The modified SET4 assay was more precise for measuring single transcripts (Rho 0.966 vs 0.888, *p*< 0.01) but not multigene expression signatures SET_ER/PR_ (Rho 0.985 vs 0.968) or PI3Kges (Rho 0.985 vs 0.946) in FFPE, compared to FF samples. It was also more precise than wtRNAseq of FFPE for measuring transcripts (Rho 0.986 vs 0.934, *p*< 0.001) and SET_ER/PR_ (Rho 0.993 vs 0.915, *p*=0.004), but not PI3Kges (Rho 0.988 vs 0.945, *p*=0.051). Accuracy (Bias) was comparable between protocols. Two samples carried a *PIK3CA* mutation, and measurements of transcribed mutant allele fraction was similar in FF and FFPE samples and appeared more precise with the modified SET4 assay. Amplification efficiency (reads per UMI) was consistent in FF and FFPE samples, and close to the theoretically expected value, when the library size exceeded 400,000 aligned reads.

**Conclusions:**

Modifications to the targeted RNAseq protocol for SET4 assay significantly increased the precision of UMI-based and reads-based measurements of individual transcripts, multi-gene signatures, and mutant transcript fraction, particularly with FFPE samples.

**Supplementary Information:**

The online version contains supplementary material available at 10.1186/s12885-021-07814-8.

## Background

Although most gene expression signatures are currently based on RT-PCR amplification or direct hybridization to oligonucleotide probes [1], targeted RNA sequencing (RNAseq) is developing into an alternative technology for translational research and clinical testing [2, 3]. Previously, we demonstrated that the quality of gene expression quantification by whole transcriptome or targeted RNAseq measurement did not differ significantly when using three different methods (kits) to purify RNA from formalin-fixed paraffin-embedded (FFPE) samples [4, 5]. Such promising analytical performance compels development of evidence-based standard operating procedures for clinical-level implementation of RNAseq on routine FFPE samples. It also raises the possibility of whether the introduction of unique molecular identifiers (UMIs), also known as molecular barcodes, during reverse transcription could help reduce the inherent variability in amplification of target RNA sequences from FFPE tissue samples by enabling more precise enumeration of sequencing reads according to the source mRNA molecules instead of the total read counts. Thus, we modified our previously published targeted RNAseq assay that is intended to predict sensitivity to endocrine-based treatments from breast cancer samples, in order to evaluate this potential improvement in transcript quantification [5, 6].

Endocrine-based therapies have a principal role in the treatment of primary and metastatic breast cancer that is estrogen receptor-positive and HER2-negative. As endocrine resistance can be acquired there is also a need to predict sensitivity to treatment during the course of Stage IV disease to potentially direct patients to alternative treatment approaches if available. We have reported that patients stratified as endocrine sensitive based on the targeted RNAseq assay for SET_ER/PR_ index (of hormone receptor-related transcription) had longer survival when they received endocrine therapy as their next treatment, and that was independent from clinical-pathologic risk factors or detection of activating mutation in the *ESR1* gene [6]. Activating *PIK3CA* mutations are also associated with greater benefit from *PIK3CA* inhibitor alpelisib [7], and with letrozole compared with tamoxifen as endocrine therapy [8]. Hence, we included a 10-gene transcriptional signature (PI3Kges) of Sinn et al that was curated from the published 287-gene microarray-based signature of transcription related to PI3K activation due to activating mutations in *PIK3CA* [9]. The SET4 targeted RNAseq assay also measures the expression of two multi-gene signatures (SET_ER/PR_ index and PI3Kges), single genes (*ESR1*, *PGR*, *ERBB2*, *AURKA*, *FGFR1*) and the proportion of transcripts carrying known hotspot mutations in *ESR1* (ligand-binding domain), *PIK3CA* (helical and loop domains), *AKT1* (pleckstrin homology domain), PTEN (phosphatase tensin-type domain), and *ERBB2* (protein kinase domain). Essentially, SET4 assay combines measurements of the genotype and phenotype related to clinically relevant oncogenic pathways in hormone receptor-positive breast cancer (Supplementary Table 1).

Targeted RNA sequencing (targeted RNAseq) of RNA transcripts of interest provides increased sensitivity, dynamic range, reduced cost, and increased throughput compared to standard sequencing of the whole transcriptome (wtRNAseq) [10]. Although the method is compatible with RNA derived from either FF or FFPE tissue samples [11] and shows good concordance between sample types, we had observed a linear bias with higher SET_ER/PR_ index measured from wtRNAseq, compared to the targeted RNAseq SET4 assay [5]. In this study, we updated our methods for performing the SET4 assay, including introduction of UMIs, and demonstrate the resultant increase in technical reproducibility, robustness to preanalytical and analytical conditions, and reduction of inter-platform bias between targeted and wtRNAseq.

## Methods

### RNA extraction from tissue samples

We used residual RNA from the same samples as previously reported [5]. Briefly, those were surgically resected tissue samples collected from 12 treatment-naïve, stage I-III invasive breast cancers (IRB protocol LAB08–0824), homogenized [12], and split for processing into fresh frozen (FF) and FFPE conditions [5]. RNA was extracted from thawed FF samples using RNeasy Mini Kit (Qiagen, Hilden, Germany) [4, 12]. RNA was extracted from adjacent FFPE tissue-block sections using FFPE RNA purification Kit (Norgen, Thorold, Canada), which we had shown to be comparable to two other commercial kits [5]. Two replicate RNA extractions for each sample were performed in parallel for both FF and FFPE. Both the FF and FFPE RNA isolation protocols used DNase I treatment. RNA concentration was quantified by Nanodrop (Nanodrop Technologies, Wilmington, DE). An additional ethanol precipitation step was applied to further clean RNA with A260/230 below 1.0.

### RNA sequencing

RNA samples underwent wtRNAseq and targeted RNAseq for 18 SET_ER/PR_ index transcripts, 10 PI3Kges transcripts, 6 well-known breast cancer mutation hotspots (in *ESR1*, *PIK3CA*, *AKT1*, *PTEN*, and *ERBB2*), 10 control transcripts, and transcripts for *FGFR1, AURKA*, *PGR*, and *ERBB2* (Supplementary Table 1). The transcript of one gene, *NAT1*, is shared between the SET_ER/PR_ index and PI3Kges. For each of the 12 breast cancer tissue samples, two technical replicates were performed for both FF and FFPE RNA, using the wtRNAseq and the two targeted RNAseq protocols (OLD and NEW). We performed at least six replicates from the two tumors with mutation detected, to further examine the mutation detection reproducibility.

wtRNAseq libraries were prepared using the RNA HyperPrep kit with RiboErase (HMR) (Kapa Biosystems, Wilmington, MA), as previously described [4]. Sequencing was performed using Illumina HiSeq 4000 (Illumina, San Diego, CA), with 6 libraries pooled per lane including FF and FFPE samples.

Targeted RNAseq libraries were prepared using a set of custom multiplex primers including an approximately 20-nucleotide (20-nt) gene specific sequence and either a 17-nt Illumina adaptor (5′-CGCTCTTCCGATCTCTG-3′) on the 5′-end of the forward primer or a 17-nt Illumina adaptor (5′-TGCTCTTCC-GATCTGAC-3′) on the 5′-end of the reverse primer.

The original reads-based targeted RNAseq sequencing libraries without UMIs (OLD_reads) were prepared using a one-step RT-PCR protocol as described previously [5]. Purified RNA was mixed with a pool of custom primer pairs, droplet stabilizer (BioRad, Hercules, CA), and SuperScript III reagents (ThermoFisher Scientific, Waltham, MA) to prepare a reaction master mix. Droplet-generation was performed using RainDance Source system and followed by a one-step RT-PCR reaction (RT at 55 °C for 30 min, then 1st PCR with 55 amplification cycles) carried out within the droplets. The droplets were destabilized and the released PCR products were purified using SPRIselect magnetic bead (Beckman Coulter, Brea, CA). A 10-cycle 2nd PCR was performed using PlatinumTaq High Fidelity DNA Polymerase (ThermoFisher Scientific, Waltham, MA) to incorporate RainDance DirectSeq primers for sample indexing and Illumina specific adapters for cluster generation/sequencing. After bead purification and Bioanalyzer quantification, the indexed libraries were pooled and sequenced on the Illumina Miseq platform (Illumina, San Diego, CA) with paired-end 215 bp reads using v3 chemistry reagents.

The UMI-based targeted RNAseq sequencing libraries (NEW_UMIs) were prepared by making the following adjustments to the ‘OLD_reads’ protocol described above. Purified RNA was reverse-transcribed at 55 °C for 10 min using SuperScript IV (ThermoFisher Scientific, Waltham, MA) and 8-nt UMI-containing primers designed as follows: 5′-Illumina adaptor sequence-NNNNNNNN-reverse primer sequence-3′, and synthesized by Integrated DNA Technologies, San Diego, CA. The resultant cDNA was purified using SPRIselect beads to remove excess primers. The cDNA, gene-specific forward primers, a universal reverse primer (5′-TGCTCTTCC-GATCTGAC-3′), and droplet stabilizer, were added to TaqMan Genotyping Master Mix (ThermoFisher Scientific, Waltham, MA), and droplets were generated using the RainDance Source system. Then a 40-cycle droplet PCR (1st PCR) was carried out to amplify the cDNA. After that, purification, 2nd PCR, library quantification and sequencing were performed as described above, with the following addition: pooled libraries were treated with Illumina Free Adaptor Blocking reagent prior to sequencing to minimize potential index hopping levels and to enhance data quality.

### Pre-processing of sequencing reads, alignment and quantification

Processing of wtRNAseq reads was performed as previously described [5]. Two custom pipelines were developed to perform read-based and UMI-based analyses of the targeted RNAseq data. For read-based analysis, blastn was used to map the paired-end reads to a custom cDNA reference including only the targeted genes [13]. The mapping results were processed by in-house Perl scripts. Reads with matched region shorter than 90 nt (the insert size is between 150 and 200 bp) or identity percentage less than 80% were filtered out. Transcript levels were quantified by counting the total number of aligned reads on each target. SET_ER/PR_ and PI3Kges were calculated as described before [4]. A single nucleotide mutation was reported if sequencing depth at a genomic position was greater than 100, the allele frequency (AF) was greater than 5%, and the ratio of R1 and R2 reads was between 0.8 and 1.2.

For UMI-based analysis, after reads were aligned and filtered as described above, the nucleotides between the 9th and 25th (gene specific reverse primer sequence) from the reverse read (R2) were extracted to identify each target gene. The first 8 nucleotides were extracted from the R2 reads as a unique molecular identifier (UMI). Reads with unmatched nucleotide within the 17-nt gene specific sequence or irregular length of UMI were excluded from further analysis. Each target has certain number of UMI groups (distinct transcripts), and each group may have one or more reads (PCR duplicates). If a UMI group contained 2% or less of the reads in the most frequent UMI group for that target, then it was removed from further analysis. Reads in the same UMI group were considered PCR duplicates and one read was randomly selected as the representative sequence for each UMI. Then the corresponding forward reads (R1) were pulled out based on the name of R2 reads. This small set of reads, corresponding to filtered unique reverse-transcribe-able mRNA species in the sample, was used for downstream analyses instead of total reads. Transcript level quantification and mutation analyses were processed as described above in the read-based analysis. An additional criterion was required for the mutation calls: sequencing depth at a position had to be at least 10 unique UMIs, roughly equivalent to 80–100 reads.

### Statistical analysis

Pearson correlation coefficient (r) was used to compare gene expression levels and transcriptional signature scores between FF and FFPE samples, and between wtRNAseq and targeted RNAseq assays. Measurements from technical replicates were averaged prior to analyses. Agreement was also assessed using Lin’s concordance correlation coefficient (CCC) [14]. Lin’s coefficient modifies the Pearson correlation coefficient by assessing not only how precisely scattered data approximate the line of best fit (Correlation term ranging from − 1 to 1; higher is better) but also accuracy, measured as how far that line is from perfect agreement (Bias term ranging from 0 to 1; higher is better). Nemenyi’s test of multiple comparisons was used to compare reproducibility between protocols. Nonparametric Wilcoxon one-sample test and Mann-Whitney U-test were used to compare average of obtained results (e.g. CCC value) for individual gene measurements between protocols. Levene’s test was used to compare variances of obtained results (e.g. CCC value) for individual gene measurements between protocols. Significance level was set to 0.05 in all analyses.

## Results

### Reproducibility of gene expression measurements

**Fig. 1:**
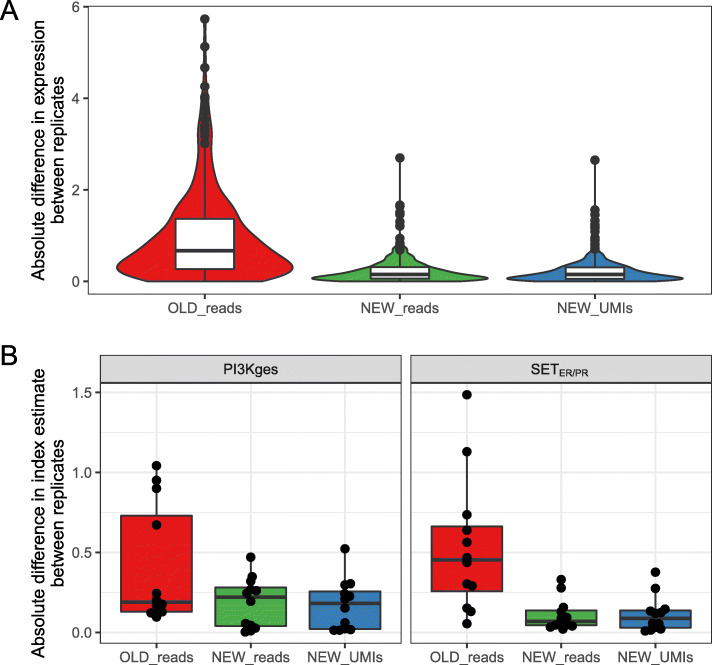
Technical variance of expression measurements from targeted RNAseq data on FFPE samples. **a** Distribution of absolute difference in expression level between replicates for 28 targeted genes within each protocol. **b** Distribution of absolute difference in molecular signature score between replicates for two signatures within each protocol. Panel B shows boxplots, rather than violin plots, due to smaller sample size in that analysis (*n*=12 vs *n*=324)

The median absolute difference of expression measurements (using total read counts) between technical replicate assays using RNA extracted from FFPE was significantly lower using the ‘NEW’ UMI-based protocol than with the original protocol (NEW_reads vs OLD_reads, *p*-value < 0.001) for individual transcripts (Fig. 1a and Supplementary Table 2) and both multi-gene signatures (Fig. 1b and Supplementary Table 2). A similar result was observed when comparing protocols with RNA from FF samples (Supplementary Fig. 1 and Supplementary Table 2), but the absolute difference was less due to less variable measurements from FF RNA [5]. Collapsing the total reads counts to UMI-based counts (NEW_reads vs NEW_UMIs) did not change the variance for measurements of individual transcripts (*p*-value = 0.94) or multi-gene signatures (*p*-values = 0.94, 1.00) (Supplementary Table 2). This suggests that the adjustments incorporated into the NEW_reads protocol removed most of the bias introduced by the PCR steps without requiring the use of UMIs. Raw read depth for individual genes was similar between protocols and was more reproducible between technical replicates using the NEW_UMIs protocol in both FF (Supplementary Fig. 2) and FFPE samples (Supplementary Fig. 3).

### Distribution of UMIs

**Fig. 2:**
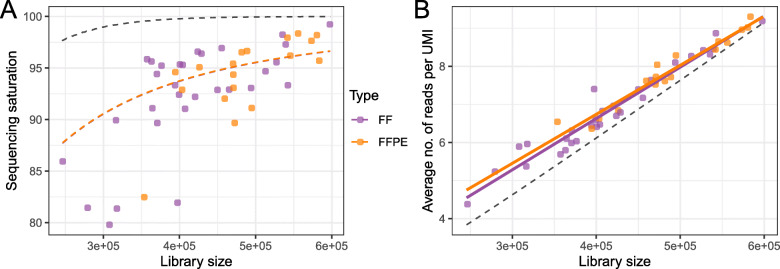
Sequencing saturation for gene quantification. **a** Percent of all possible UMIs observed in a sample (sequencing saturation) as a function of the total number of aligned reads (library size). The black dotted line shows the theoretical sequencing saturation assuming no PCR bias or sequencing errors in UMIs (binomial distribution). The colored dotted lines show sequencing saturation modelled by a negative binomial distribution fitted to the observed values, separately for each sample type. One hundred percent means 65,536 unique UMIs. **b** Association of the average number of reads per UMI and total number of aligned reads (library size). The black dotted line shows the theoretical relationship based on the binomial distribution. Colored solid lines show linear regression fitted to observed values for each sample type

We compared the percentage of all possible UMI sequences (65,536 different UMIs for 8 nt sequences) that were observed in a single library (sequencing saturation) against the total number of aligned reads (library size), for targeted RNAseq libraries from both FF and FFPE samples (Fig. 2a). For comparison, we included idealized theoretical sequencing saturation curves based on the negative binomial distribution that assumes no bias from PCR or other factors, e.g. sequencing errors in UMIs (dashed lines). There was only a very small difference observed (< 5%) between the ideal and fitted distributions for libraries larger than 400,000 aligned reads.

The negative binomial distribution is a suitable model for sequencing read distributions because the modelled variance captures between-sample variance [15]. Our observed distributions of UMIs were well represented using negative binomial distribution, suggesting that measured reads were relatively free from additional introduced bias or noise. We observed that the average number of reads per UMI was close to the theoretical expected value (dashed line) for libraries larger than 400,000 aligned reads (Fig. 2b). The overlap of FF and FFPE distributions seen suggests that the NEW protocol had negligible additional PCR bias from cross-linking of RNA in FFPE samples (Fig. 2b).

### Concordance between sample types and platforms

**Fig. 3:**
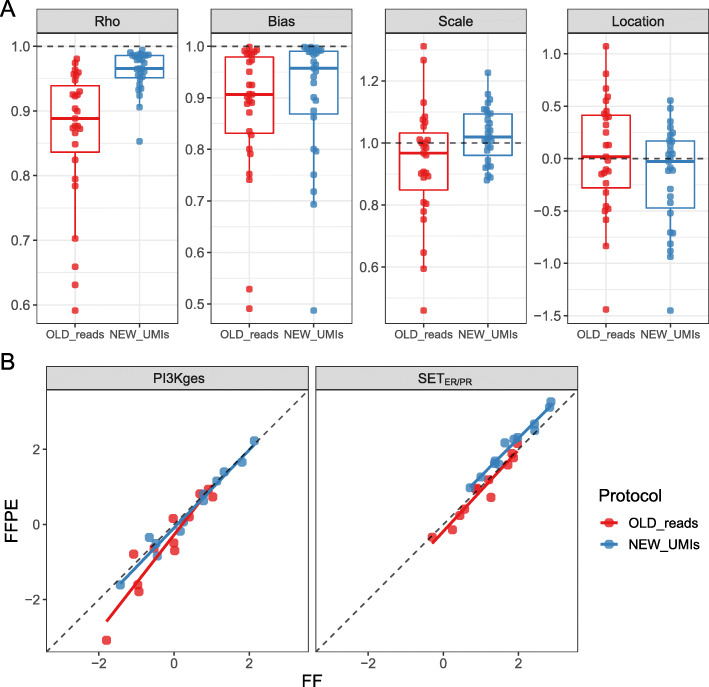
Concordance of gene expression measurements between FFPE and FF samples from the targeted RNAseq assays. **a** Distribution of the components of the concordance correlation coefficient (CCC), Pearson correlation coefficient (Rho), overall bias, scale bias and location bias, for the 28 signature genes within each protocol. **b** Concordance for the two transcriptional signature scores for two protocols

The concordance of individual transcript measurements between matched FF and FFPE samples was greater with the NEW_UMIs protocol, exhibiting significantly higher Rho (median 0.966 vs 0.888 for NEW_UMI vs OLD protocols; *p*-value < 0.001) but similar bias (median 0.958 vs 0.907 for NEW_UMI vs OLD protocols; *p*-value = 0.53) (Fig. 3a and Supplementary Table 3). Both multi-gene signatures were strongly concordant between matched FF and FFPE samples, SET_ER/PR_ (CCC 0.901, 95%CI 0.766–0.960) and the PIK3CA-related PI3Kges (CCC 0.982, 95%CI 0.941–0.994), representing a significant improvement over the OLD protocol for the PI3Kges (CCC 0.861, 95%CI 0.680–0.943), but not significantly different for SET_ER/PR_ (Table 1 and Fig. 3b). The NEW_UMIs protocol appears to have shifted the location of both signatures, correcting lower FFPE scores in PI3Kges, but introducing a small location shift bias in the SET_ER/PR_ index (Fig. 3b). Measurements of both multi-gene signatures had minimal scatter (Rho 0.985, Table 1).

**Table 1:** Concordance of the two BC signatures, and gene expression, between matched FFPE and FF samples. Gene Expression refers to individual gene measurements, for which values in the table are medians over all genes. The combination of protocol (NEW vs OLD) and data summarization of mapped counts (UMI groups vs reads). Bold font indicates higher value for given signature

Signature	Protocol	CCC	CCC low CI	CCC high CI	Rho	Bias	Scale	Location
PIK3CA	OLD_reads	0.861	0.680	0.943	0.946	0.910	0.729	0.313
	NEW_reads	0.980	0.934	0.994	0.984	0.995	**0.956**	0.089
	NEW_UMIs	**0.982**	0.941	0.994	**0.985**	**0.996**	0.950	**0.072**
SET_ERPR	OLD_reads	0.956	0.871	0.985	0.968	0.987	0.881	**0.103**
	NEW_reads	**0.901**	0.764	0.960	0.984	**0.916**	0.953	−0.425
	NEW_UMIs	**0.901**	0.766	0.960	**0.985**	0.915	**0.977**	−0.432
Gene Expression	OLD_reads	0.785	–	–	0.888	0.907	0.967	0.018
	NEW_reads	0.911	–	–	**0.977**	0.951	**0.997**	**−0.015**
	NEW_UMIs	**0.921**	–	–	0.966	**0.958**	1.019	−0.027

There was strong concordance between targeted RNAseq and wtRNAseq measurements of individual transcripts in both FF and FFPE samples (Supplementary Table 4 and Supplementary Fig. 4). That correlation was significantly improved with the NEW protocol for both FF and FFPE samples, and included a decrease in location bias with FFPE samples (Supplementary Table 5; median Rho in FF 0.979 vs 0.947, median Rho in FFPE 0.986 vs 0.934 for NEW_UMI vs OLD protocols; *p* < 0.001 in both cases).

### Quantification of transcribed mutant allele fraction

**Fig. 4:**
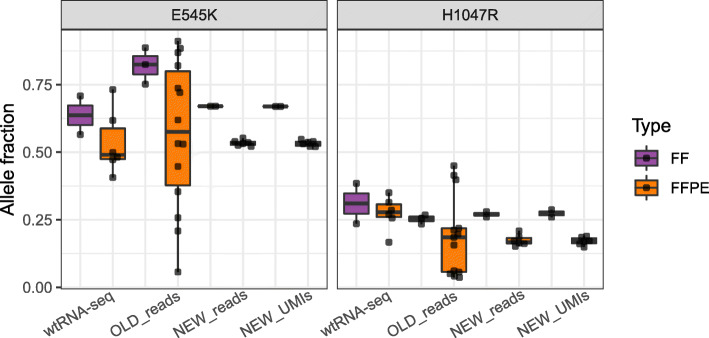
Expressed mutations estimated from targeted RNAseq reads. Distribution of the mutant allele fraction (AF) for two activating mutations in PIK3CA as quantified by each protocol and sample type

Activating mutations in *PIK3CA* transcripts were detected in two different primary cancer samples, one containing a variant (*PIK3CA* E545K) at high mutant allele fraction variant and another cancer containing a different variant (*PIK3CA* H1047R) at a lower allele fraction. These results were not adjusted for histologic tumour cell component. In both cases, we performed at least six replicate experiments for all assay conditions. The high variability in the estimated mutant allele fraction from FFPE samples using the OLD_reads protocol was lower with the NEW_reads and NEW_UMIs protocols (Fig. 4 and Supplementary Table 6). With the OLD protocol, the mutation rates varied among technical replicates (CV = 78 and 48%, respectively, for the two activating PI3KCA mutations). With the NEW protocol (NEW_reads and NEW_UMIs), the mutation rate variation was mitigated and the CV among technical replicates was reduced to approximately 10% for both mutations. We also summarize the allele fraction of the same mutations using wtRNAseq. However, due to much lower depth of coverage in the region of selected mutations on whole transcriptome platform, the variance of allele fractions was higher with wtRNAseq than from targeted RNAseq using the UMI-based protocol (Fig. 4 and Supplementary Table 6). Allele frequency calls were slightly lower from FFPE samples and were identical when calculated from total reads counts or UMI-based counts in both FF and FFPE samples (Fig. 4 and Supplementary Table 7).

## Discussion

Amplicon-based targeted RNAseq is a highly multiplexed PCR approach that relies on efficient and accurate cDNA synthesis and amplification. High performance of pooled primers, robust reverse transcriptase, and unbiased PCR amplification are critical to the success of targeted RNAseq. When working with FFPE RNA, the reverse transcription efficiency is relatively low due to RNA fragmentation and modification that occurs during FFPE fixation. With the original “OLD” protocol, we observed lower detection reproducibility in FFPE RNA compared to FF, which we assume was caused by the low efficiency and uneven sampling of RT-PCR. In other words, input FFPE RNA fragments were not equally reverse transcribed into cDNA, so were not equally represented in the sequencing data. Additionally, amplification bias might also affect the precision and accuracy of RNAseq in quantifying transcript level with FFPE tissues. Labeling individual transcripts with UMIs makes it possible to identify and correct such biases. To this end, we made three major adjustments to the OLD protocol in order to improve transcript quantitation from FFPE samples: (i) a two-step RT-PCR process replaced the one-step RT-PCR; (ii) SuperScript IV replaced SuperScript III reverse transcriptase; (iii) UMIs were attached to individual mRNA molecules during cDNA synthesis.

Although the primer pairs used in this assay were designed carefully and examined thoroughly, the cross interaction among pooled primers is unavoidable and could affect the efficiency of cDNA synthesis and PCR amplification. With the OLD one-step RT-PCR protocol, forward and reverse gene specific primers (47 pairs) were pooled together and used for both cDNA synthesis and PCR amplification. With the NEW two-step RT-PCR protocol, only gene specific reverse primers (*n*=47) were used for reverse transcription, and these primers were later removed from synthesized cDNA during purification. Then, only gene specific forward primers (n=47) and one shared reverse primer were added into the cDNA to fulfill PCR amplification. This adjustment halved the size of the primer pool and improved its working efficiency and reaction evenness by minimizing primer cross interaction. In addition, utilizing SuperScript IV in the NEW protocol significantly improved RT efficiency and evenness because of its superior robustness, processivity, and cDNA yields, compared to SuperScript III that was used in the previous protocol [16]. Adoption of the above protocol adjustments significantly improved transcript detection and reproducible quantification from FFPE samples, both for individual genes and for the transcriptional signatures, and increased the correlation in transcript levels between paired FF and FFPE samples. Most importantly, this modified protocol demonstrated great improvement in mutation detection in FFPE samples.

The protocol was adjusted in order to introduce UMIs into cDNA synthesis by using UMI-labeled gene specific reverse primers. Thus, resultant cDNA fragments are individually tagged so that each mRNA molecule that is amplified can be tracked from the original sample through the library preparation and sequencing process. At the end, we can count the total number of reads or collapse the data to count the number of unique UMIs. This approach allows identification and correction for possible PCR amplification bias [17]. Based on UMI-based quantification, we learned that greater than 90% of the library complexity has been sequenced in most cases, for both FF and FFPE samples, and the average number of reads per UMI was close to the expected value. These results indicated that the NEW protocol has been optimized properly and explained why both gene expression and mutation detection significantly improved for FFPE RNA. When the libraries were prepared using the NEW protocol, reads-based and UMI-based quantification were similar, suggesting lack of substantial amplification bias during library preparation. We believe this is largely due to utilizing Droplet PCR in the protocol, which provides superior specificity, coverage uniformity, and sensitivity relative to bulk PCR. So, efficient RT followed by droplet-based PCR of cDNA minimized PCR amplification bias in FFPE samples and thereby obviated the need for UMIs that would have helped to account for PCR amplification bias. The FFPE samples in this study were of reasonable quality (mean 2.9 RNA integrity number and 79.5% DV200), but it will also be important to determine whether incorporation of UMIs may be helpful with lower quality FFPE samples.

We note that the UMI counts from one replicate from FFPE sample 18Y were systematically lower than reads-based counts across all genes (Supplementary Fig. 3). This observation seems aberrant from the other replicate from sample 18Y and from all the other FFPE samples (Supplementary Fig. 3), and from all the FF samples (Supplementary Fig. 2). The reason for that single aberrant observation is not clear but might be post-analytical, rather than technical.

The preferred read depth varies depending on the goals of a targeted RNA-Seq study. For diagnostic purposes, higher reads depth improves accuracy and reliability of detection, especially for low-expression genes and low-frequency mutations. We note that Illumina’s recommended read depth is 3 million reads per sample for the TruSight RNA Pan Cancer (1385 targets, average of 2166 reads per target) and for the TruSight RNA Fusion Panel (507 targets, average of 5917 reads per target) [18]. For our SET4 assay, we recommend at least 400,000 reads per sample (47 targets, average of 8510 reads per target) based on our comparison with theoretical sequencing saturation curves from the negative binomial distribution (Fig. 2).

Sequencing data quality is another critical factor for successful targeted RNAseq, and the density of clonal clusters on a sequencer has a large impact on the data quality. Before loading, the pooled library was further purified to remove residual free adaptors and the loading concentration was optimized to 10pM to ensure the cluster density about 1000 K/mm^2^. As a result, all raw data created for this study were of high quality and more than 90% of sequencing runs had both Q30 and PF greater than 95%. This is crucial for the accuracy of gene expression and mutation analysis.

The targeted sequencing assay showed a slight adjustable bias compared to wtRNAseq, illustrating a systematic platform difference. These employ markedly different molecular protocols of target amplification for library preparation, even though the actual sequencing platforms are quite similar. The bias may also arise from low expressed transcripts, as most noise in the data comes from low read counts [19]. Only deeper sequencing of the transcriptome may reveal low abundance transcripts and splice junctions [20], but in many cases, it might be too costly unless a targeted approach is used. Amplification biases in targeted sequencing appear to have been adequately controlled under the NEW_UMIs protocol.

Our study was limited to only 12 cancer samples collected under a supervised research protocol and does not represent the full diversity of specimen handling and fixation methods expected across pathology laboratories or for extracting RNA and performing RNA sequencing. Also, we could not study pre-analytical effects resulting from prolonged storage of FFPE blocks prior to sectioning, as a potentially important factor in retrospective analysis of clinical trial samples.

Compared to other UMI-based RNA sequencing protocols, our method uses droplet PCR for target amplification, instead of bulk PCR. Competition between templates may be avoided by separating templates into picoliter-sized droplets and improve the coverage uniformity of sequencing and the detection sensitivity of low-copy or rare targets. During data analysis, our sequencing reads were mapped to a custom cDNA reference of 150 kilobases that only including targeted genes, whereas other methods map reads to a whole genome (3 billion bases) or transcriptome (45 million bases). Our custom reference improves mapping speed and eliminates off-target alignment. A disadvantage of our protocol is the additional time required for droplet generation (about 30 min) and the associated additional cost (about $40USD per sample).

## Conclusions

Modification of the protocol for a targeted RNAseq assay (SET4) to incorporate UMIs significantly improved the technical reproducibility of the assay, particularly for assays performed with FFPE tissue samples. Sequence saturation and average read number per UMI group were both positively correlated with library size, and were satisfactorily close to theoretical parameters. The protocol modification improved the concordance, mostly improving precision, of gene expression measurements from FFPE and FF samples, and between targeted RNAseq and wtRNAseq assays. The consistency of transcribed variant allele fraction calls between technical replicates was also improved. Importantly, proper optimization of the reverse transcription step and the use of droplet-based PCR sufficiently controlled the analytical variability from FFPE samples, such that UMI-based counts did not further improve the accuracy of measurements of gene expression and transcribed mutation fraction from these FFPE samples. However, incorporation of UMI-based analysis allowed optimization and provides additional quality control metrics for the SET4 assay.

## Supplementary Information

**Additional file 1: Fig. S1**. Technical variance of expression measurements in targeted RNAseq data using FF samples. (A) Distribution of absolute difference in expression between replicates for 28 targeted genes within each protocol. (B) Distribution of absolute difference in molecular signature score between replicates for 2 signatures within each protocol.

**Additional File 2: Fig. S2.** Distribution of raw read depth across all gene targets in FF samples. Line type (solid or dashed) distinguishes two technical replicates (6 samples have only 1 replicate in FF), while color represents different protocol. Genes are sorted by average read depth in all samples.

**Additional File 3: Fig. S3.** Distribution of raw read depth across all gene targets in FFPE samples. Line type (solid or dashed) distinguishes two technical replicates, while color represents different protocol. Genes are sorted by average read depth in all samples.

**Additional File 4: Fig. S4.** Concordance between wtRNAseq and targeted RNAseq of gene expression levels and transcriptional signature scores. Distribution of Pearson correlation (Rho), bias, scale and location coefficient (components of concordance correlation coefficient (CCC)) for all 28 targeted genes within each protocol. The green points represent the two transcriptional signatures.

**Additional File 5: Table S1**. List of genes included in SET4 assay. **Table S2**. Pairwise comparison of reproducibility (difference between replicates) between protocols. **Table S3**. Wilcoxon paired test and Levene’s test for concordance measures from comparison between FF and FFPE of individual gene measurements. **Table S4**. Concordance analysis of two BC signatures, and all the transcripts, between wtRNAseq and targeted RNAseq. **Table S5**. Wilcoxon paired test and Levene’s test for concordance measures from comparison between wtRNAseq and targeted RNAseq of individual gene measurements. **Table S6**. Summary statistics for allele fraction (AF). **Table S7**. Wilcoxon test for allele fraction (AF) comparison between FF and FFPE.

## Data Availability

The raw wtRNAseq and targeted RNAseq datasets analyzed during the current study are available from the corresponding author on reasonable request.

## Abbreviations

CCC: Concordance correlation coefficient
FF: Fresh frozen
FFPE: Formalin-fixed paraffin-embedded
nt: Nucleotides
PCR: Polymerase chain reaction
RNAseq: RNA sequencing
SET_ER/PR_: Index for sensitivity to endocrine therapy
PI3Kges: Characterizes a set of gene expression-induced changes associated with *PIK3CA* mutation
UMI: Unique molecular identifier
wtRNAseq: Whole transcriptome RNA sequencing
