# Supplementary figures and images for: Targeted RNAseq assay incorporating unique molecular identifiers for improved quantification of gene expression signatures and transcribed mutation fraction in fixed tumor samples
Source: BMC Cancer. 2021 Feb 4;21:114. doi: 10.1186/s12885-021-07814-8 (PMC7860187; doi:10.1186/s12885-021-07814-8)

A

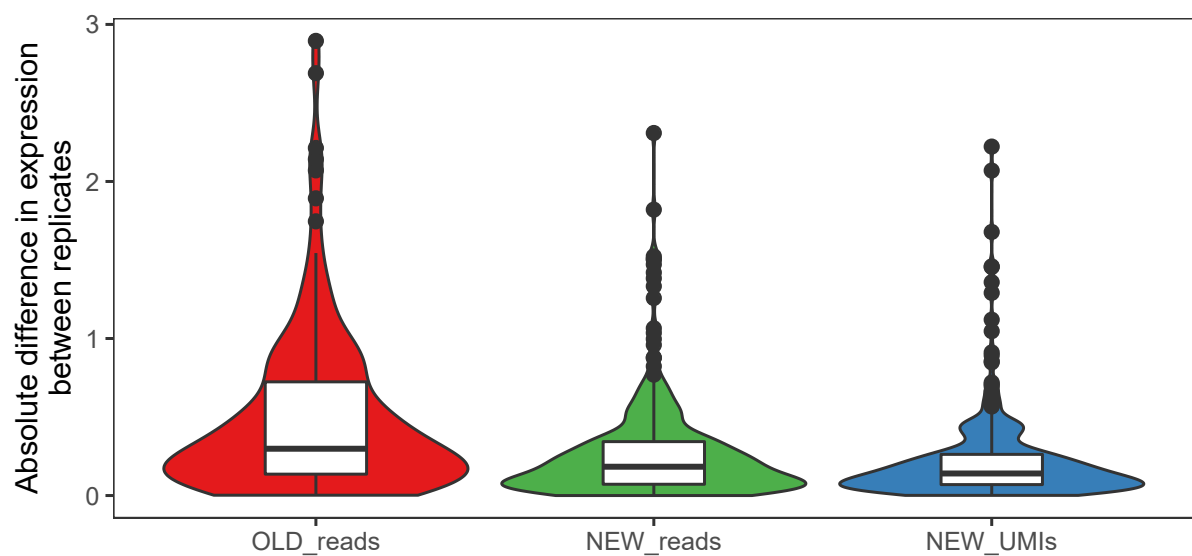

B

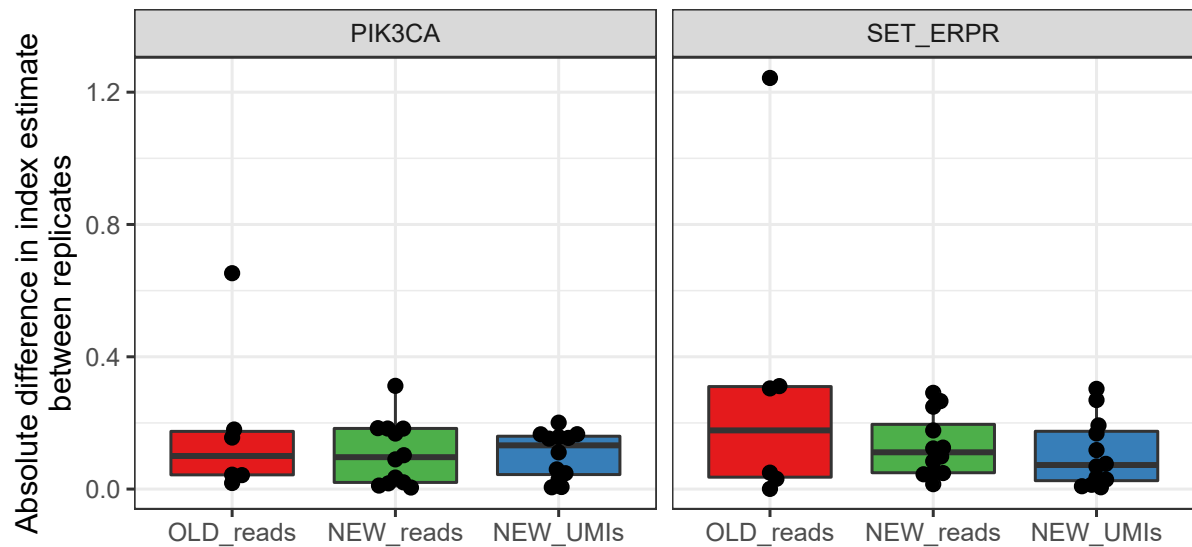

Supplement: Supplementary file 1 — Additional file 1: Fig. S1. Technical variance of expression measurements in targeted RNAseq data using FF samples. (A) Distribution of absolute difference in expression between replicates for 28 targeted genes within each protocol. (B) Distribution of absolute difference in molecular signature score between replicates for 2 signatures within each protocol. [file 12885_2021_7814_MOESM1_ESM.pdf]

Log2 read depth

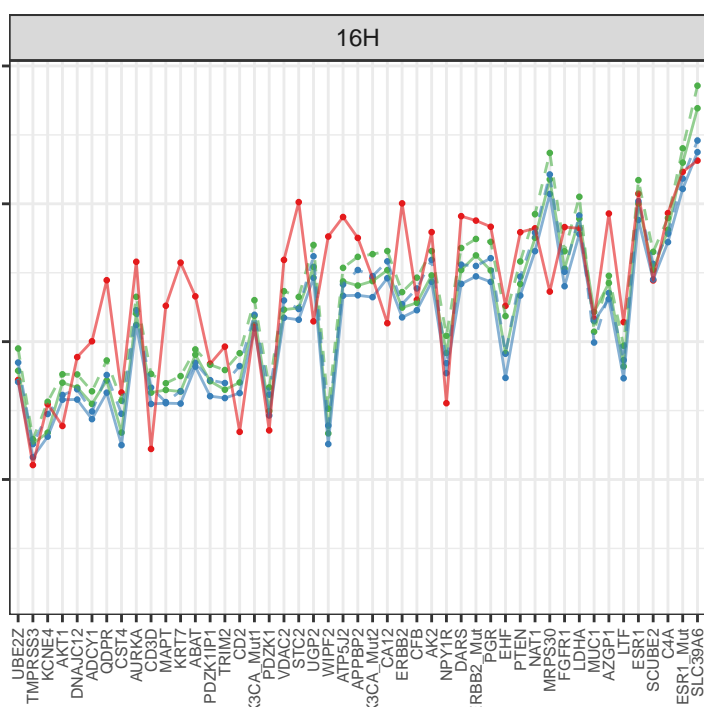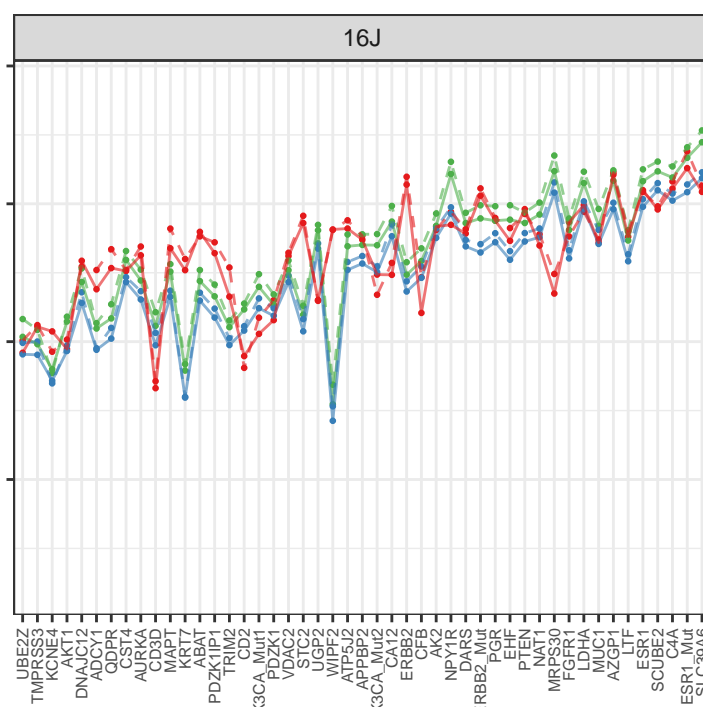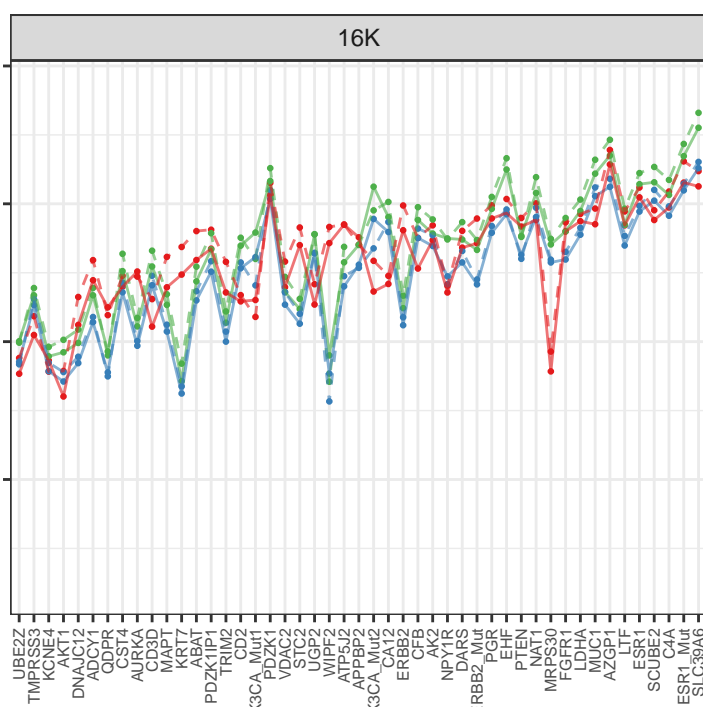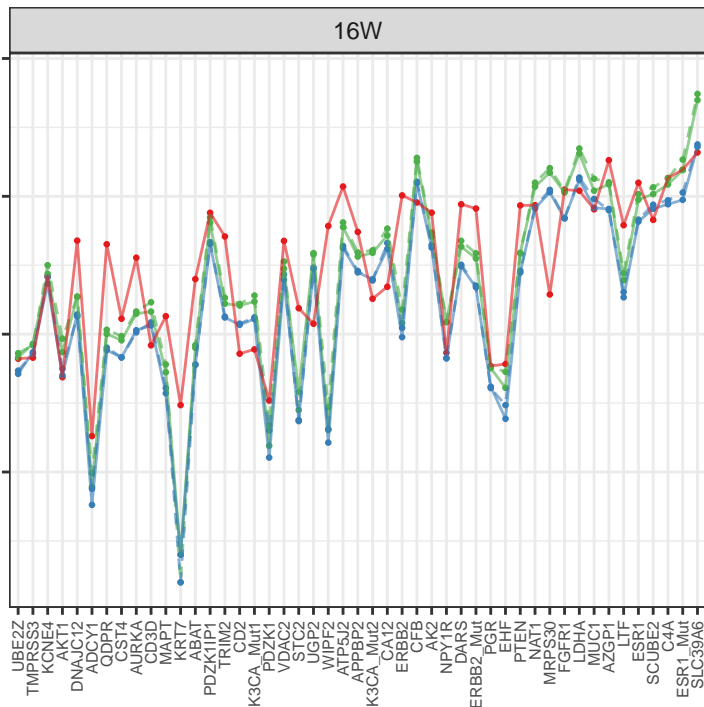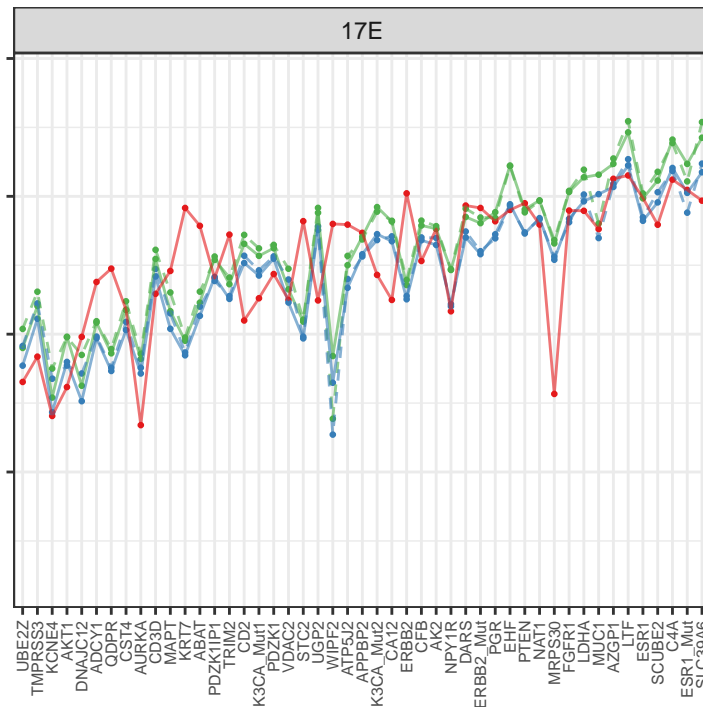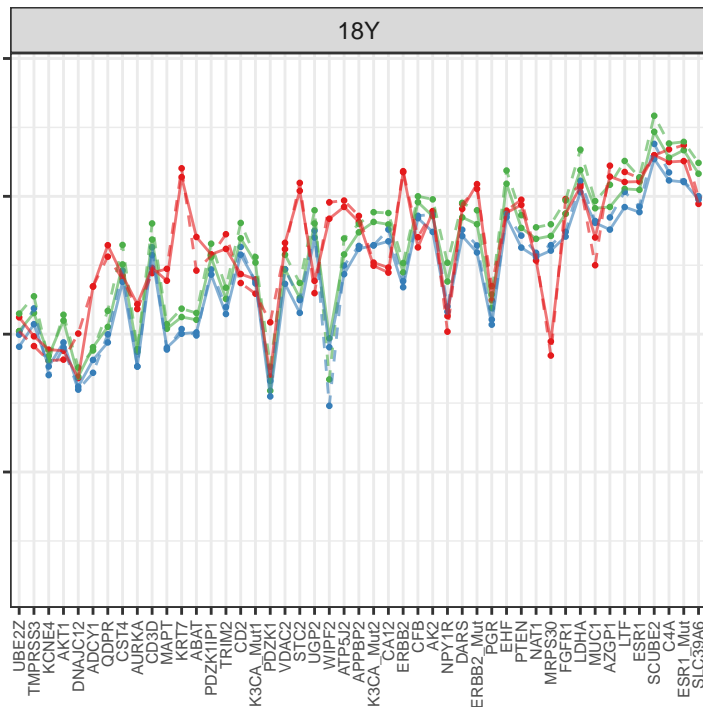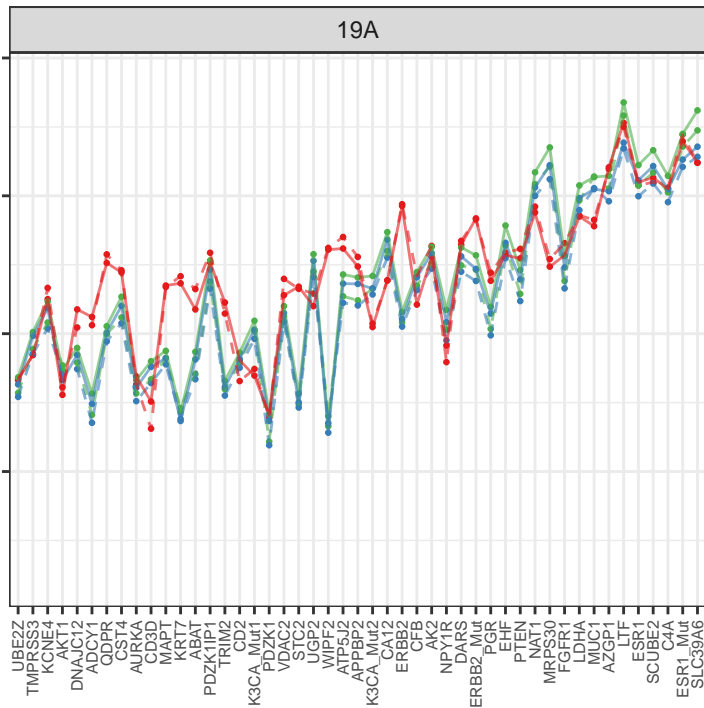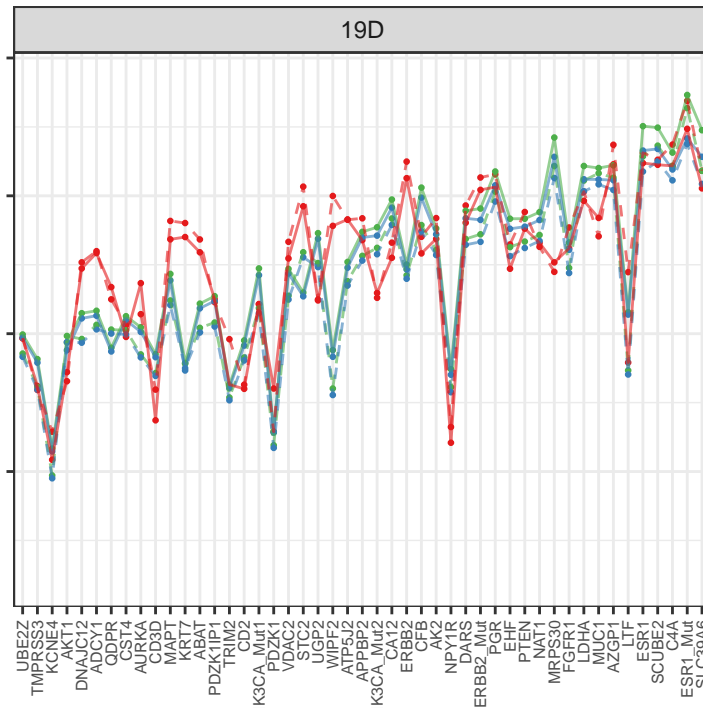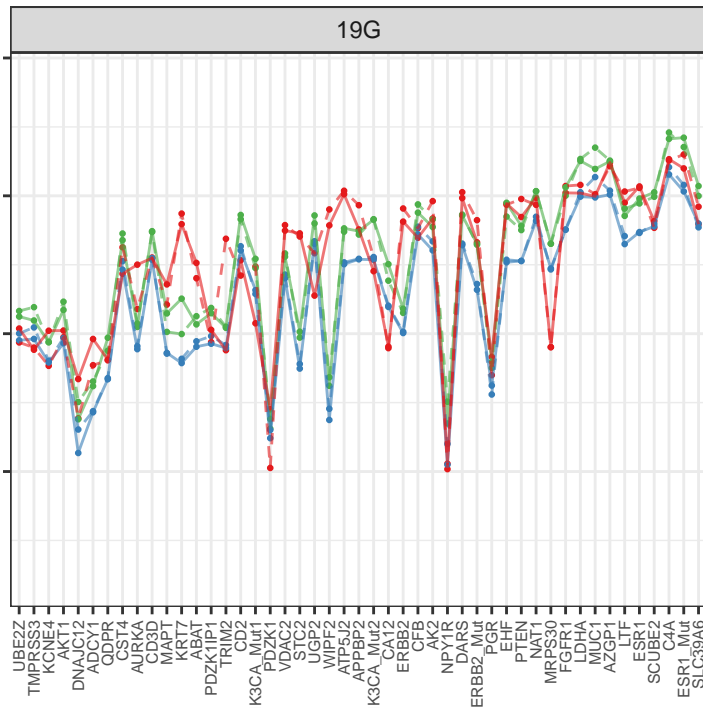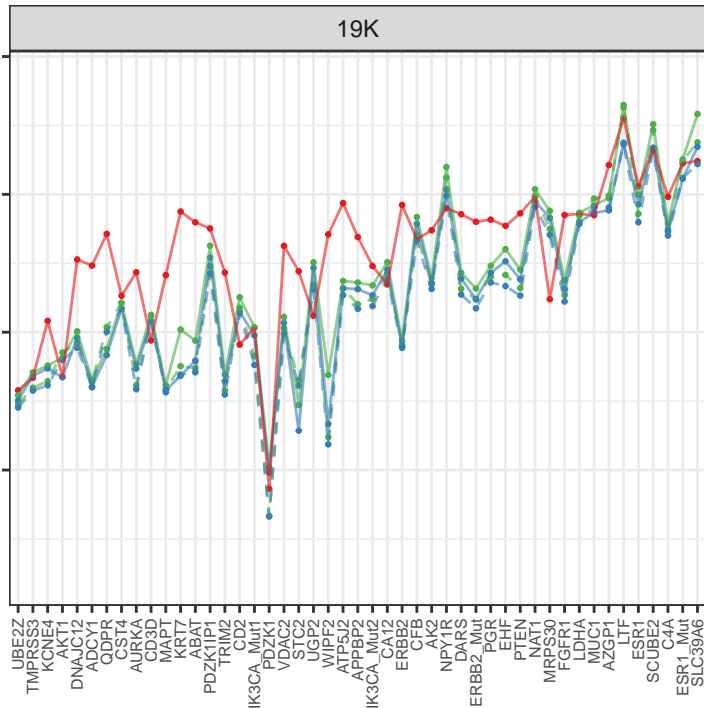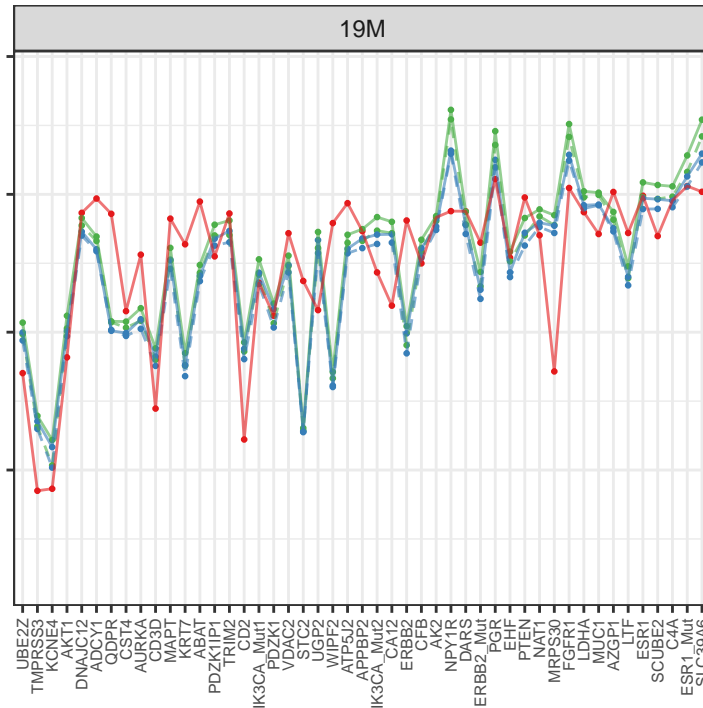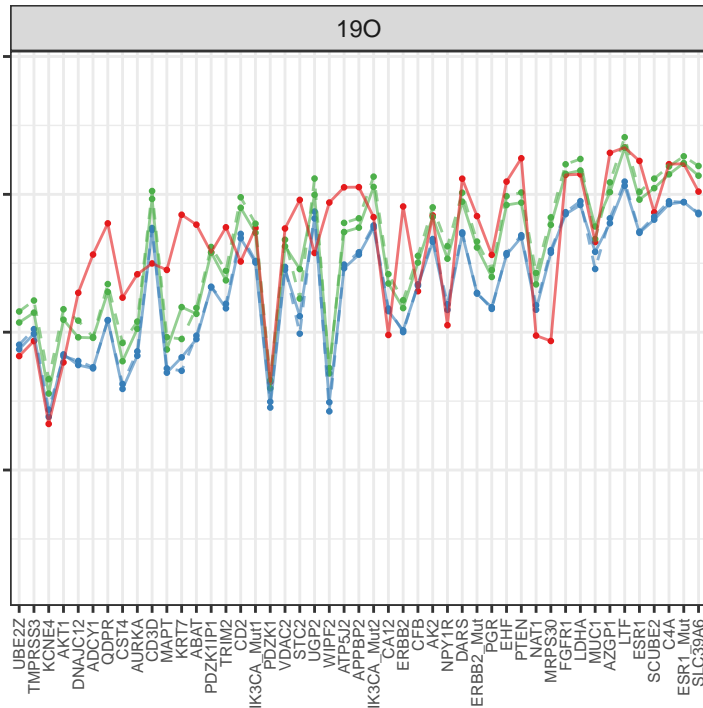

Method    • OLD\_reads    • NEW\_reads    • NEW\_UMIs

Supplement: Supplementary file 2 — Additional File 2: Fig. S2. Distribution of raw read depth across all gene targets in FF samples. Line type (solid or dashed) distinguishes two technical replicates (6 samples have only 1 replicate in FF), while color represents different protocol. Genes are sorted by average read depth in all samples. [file 12885_2021_7814_MOESM2_ESM.pdf]

Log2 read depth

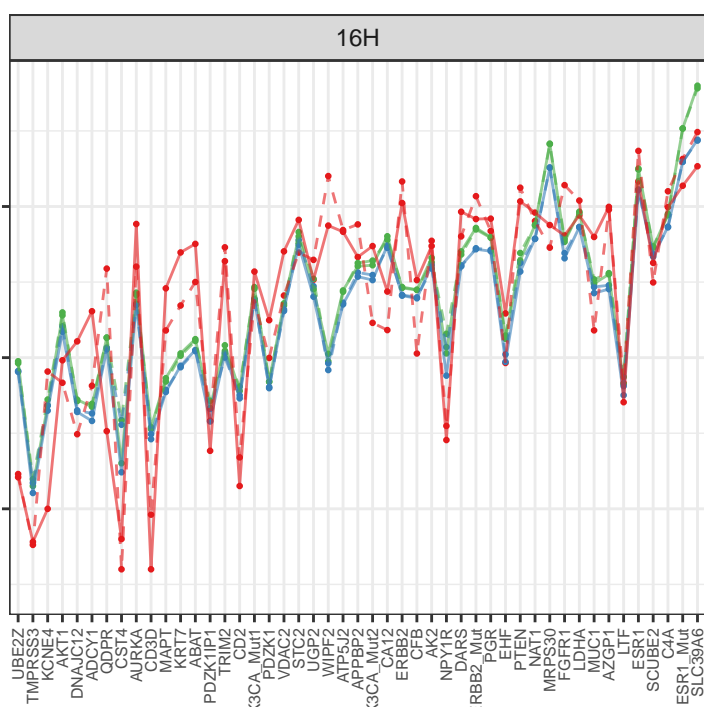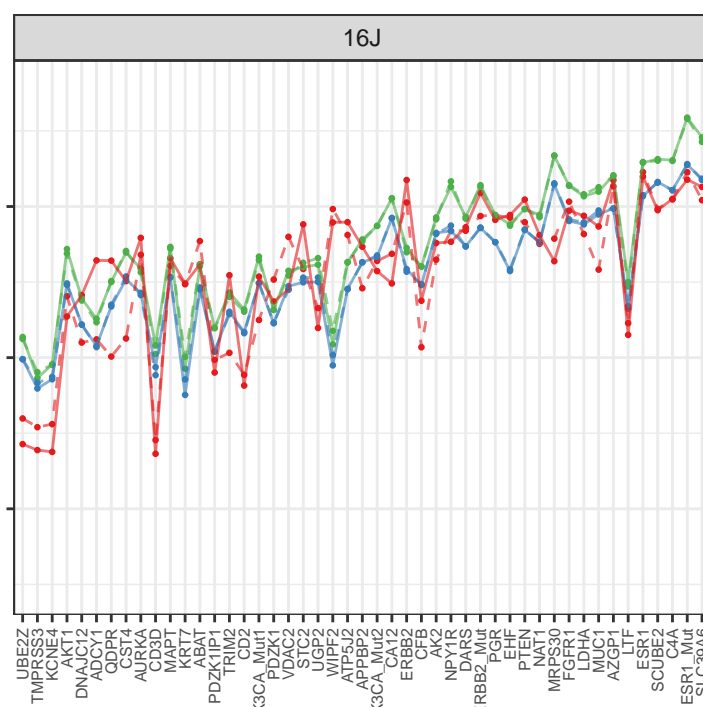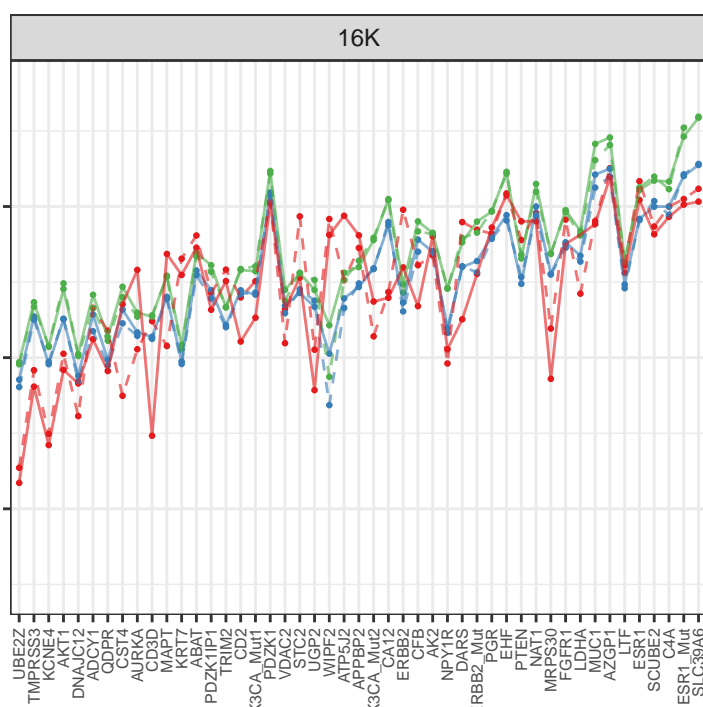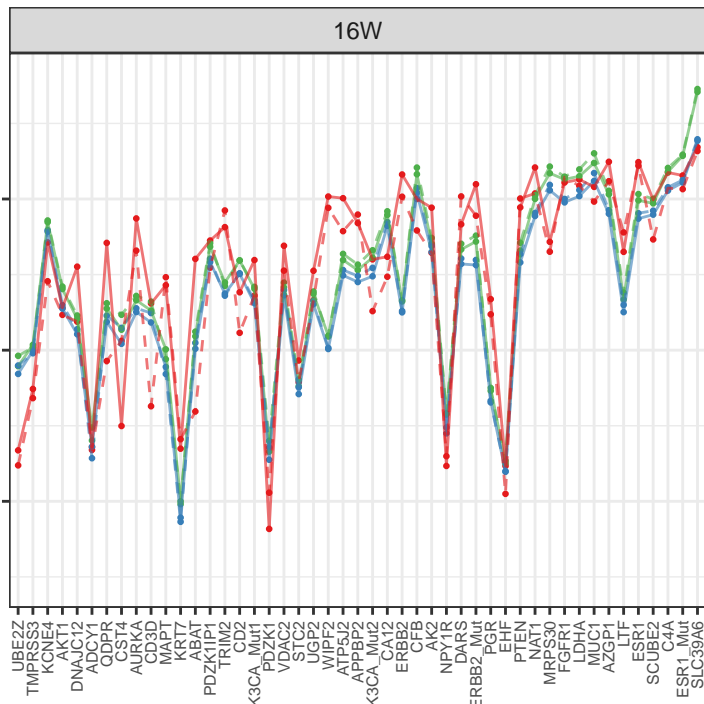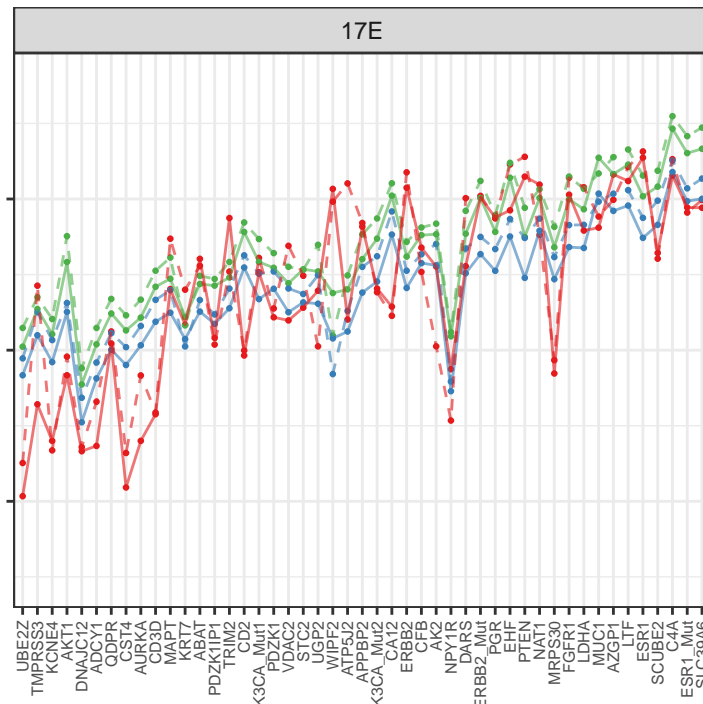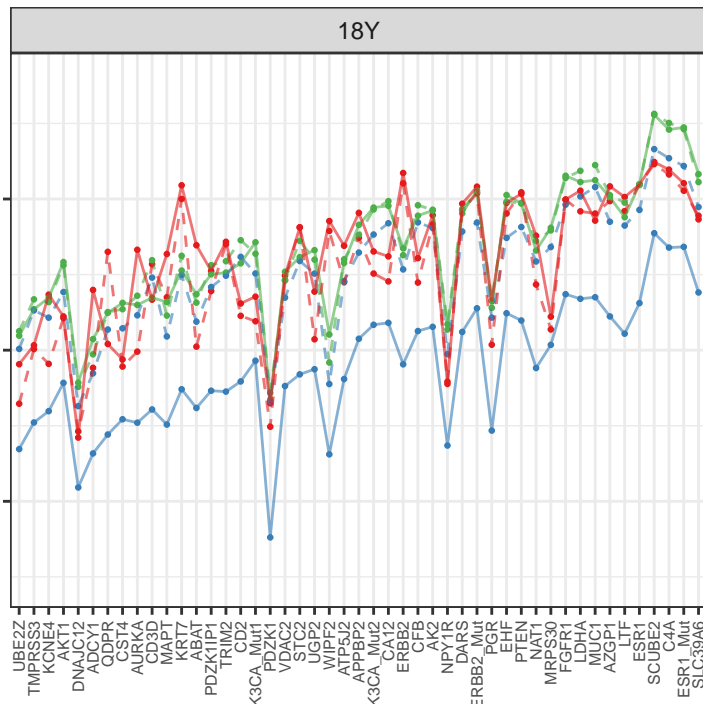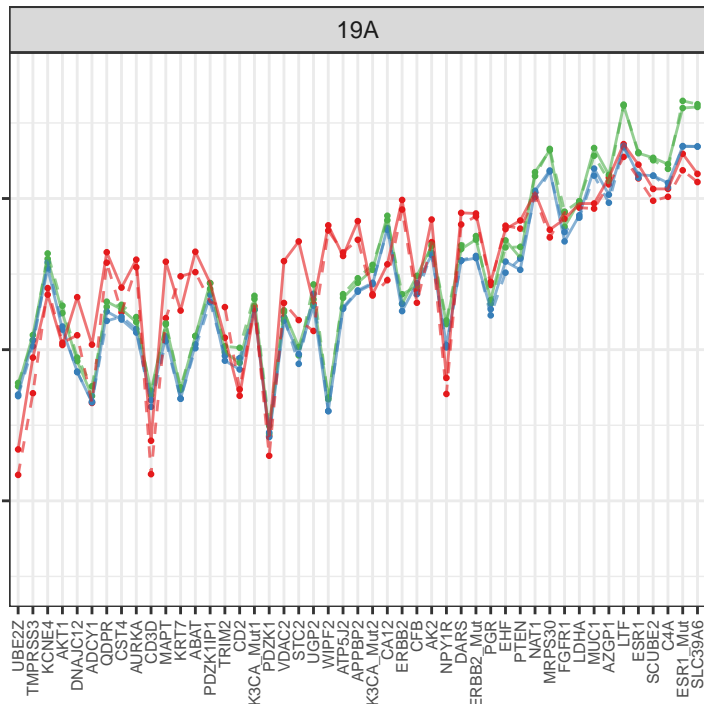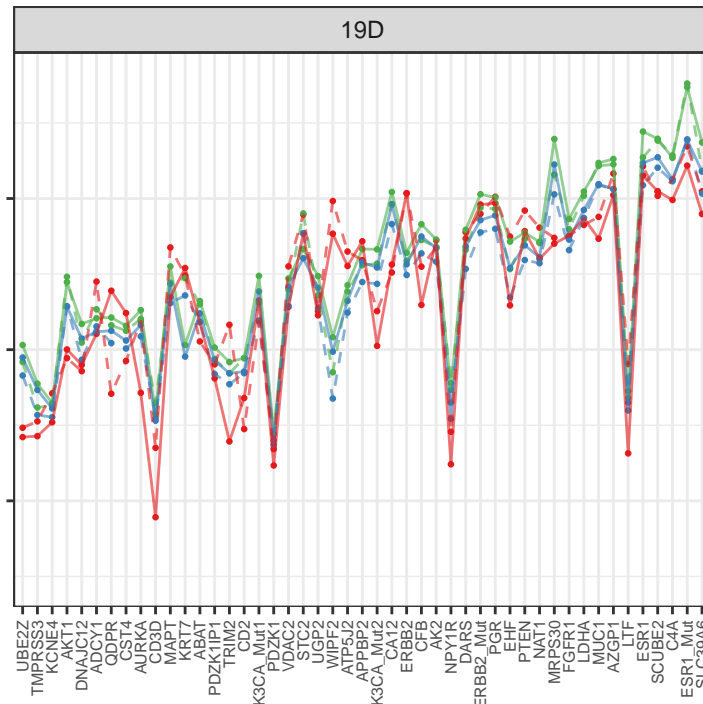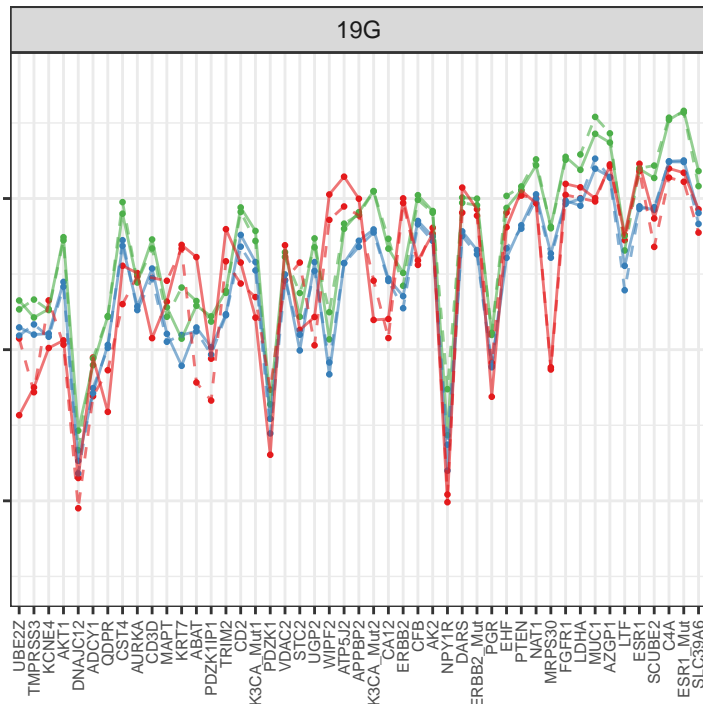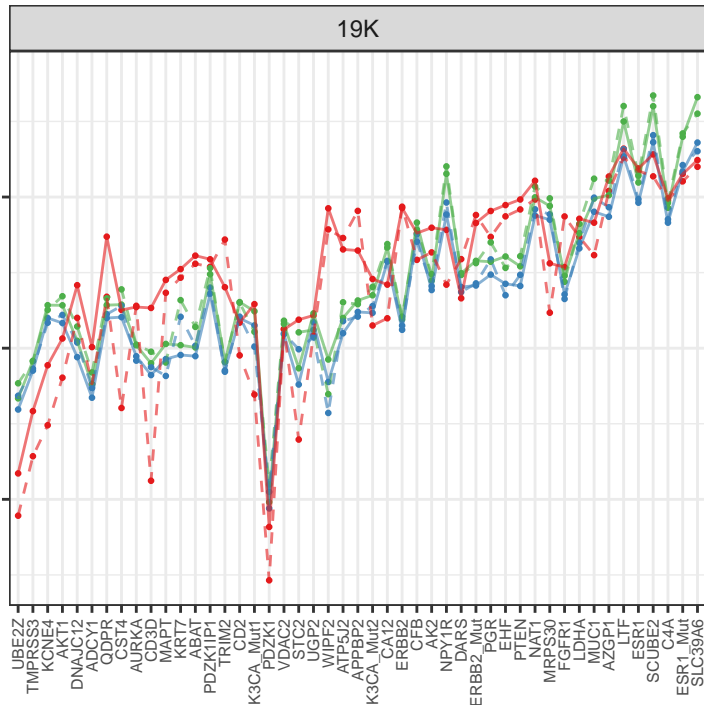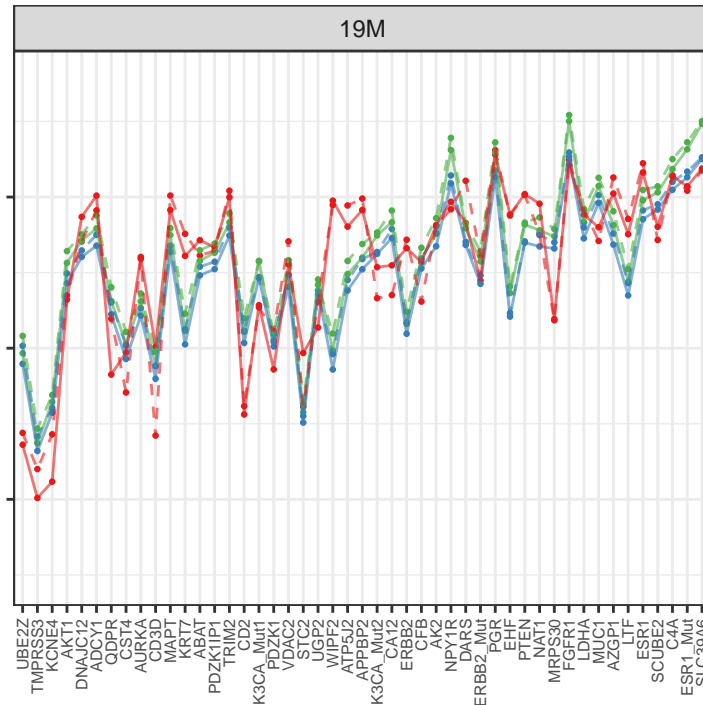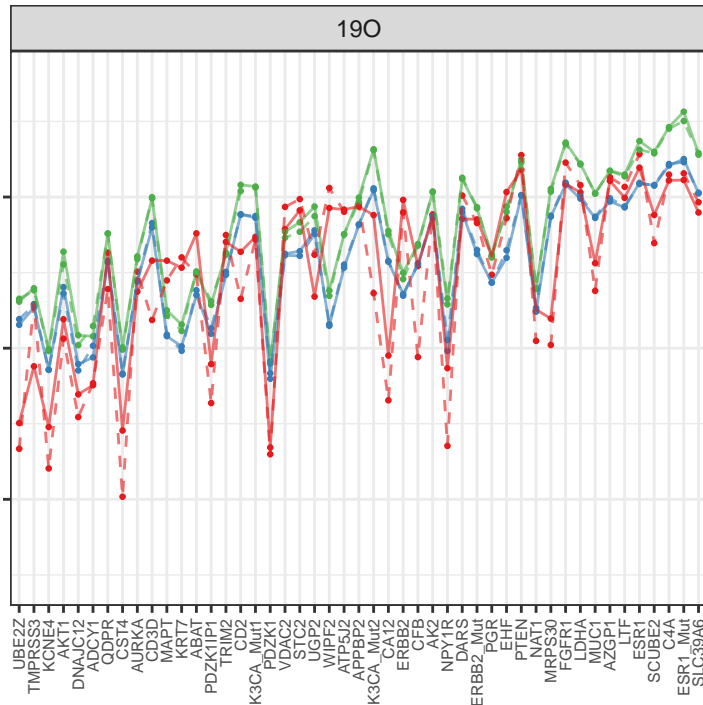

Method    • OLD\_reads    • NEW\_reads    • NEW\_UMIs

Supplement: Supplementary file 3 — Additional File 3: Fig. S3. Distribution of raw read depth across all gene targets in FFPE samples. Line type (solid or dashed) distinguishes two technical replicates, while color represents different protocol. Genes are sorted by average read depth in all samples. [file 12885_2021_7814_MOESM3_ESM.pdf]

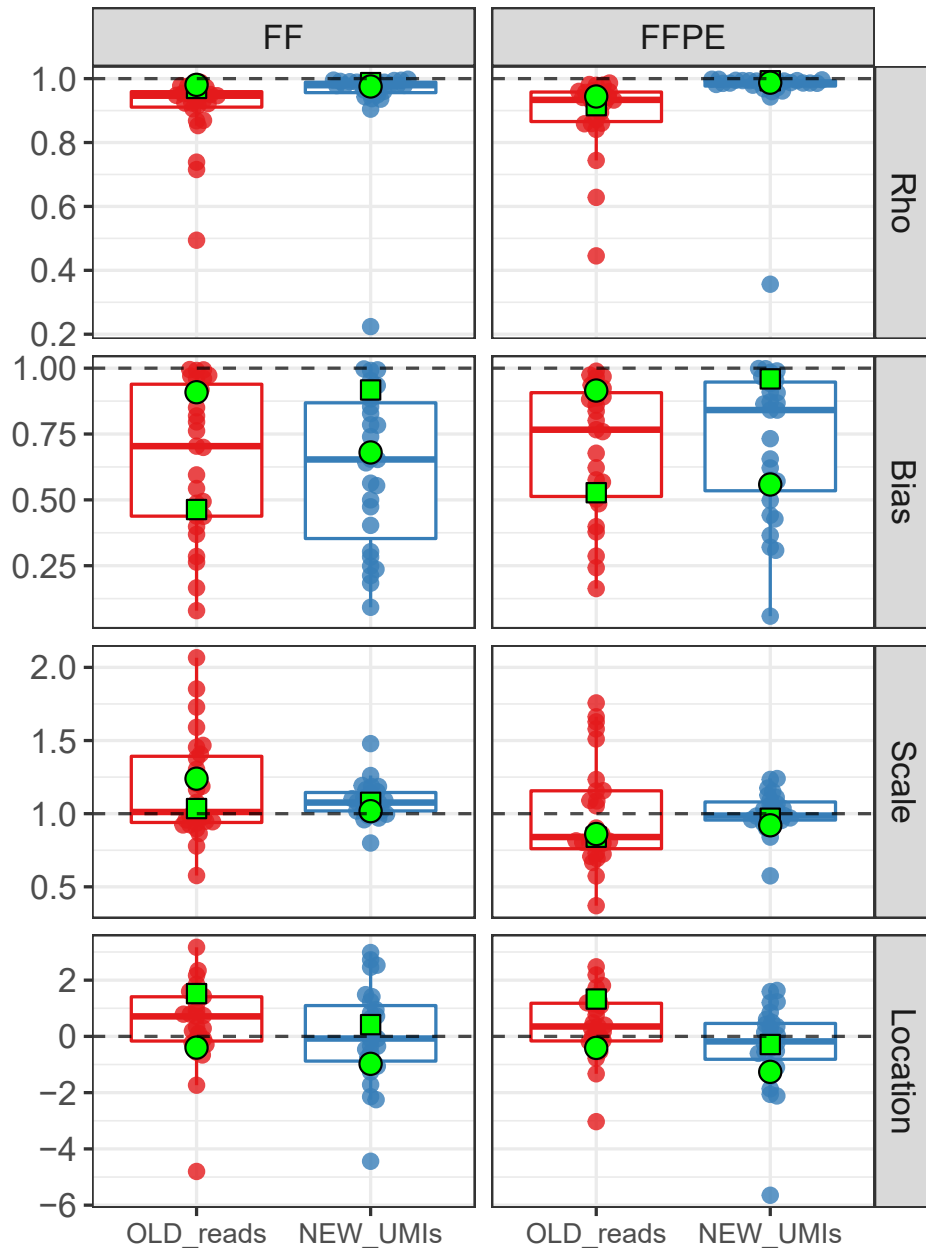

Supplement: Supplementary file 4 — Additional File 4: Fig. S4. Concordance between wtRNAseq and targeted RNAseq of gene expression levels and transcriptional signature scores. Distribution of Pearson correlation (Rho), bias, scale and location coefficient (components of concordance correlation coefficient (CCC)) for all 28 targeted genes within each protocol. The green points represent the two transcriptional signatures. [file 12885_2021_7814_MOESM4_ESM.pdf]
